# Diagnostic performance of calf circumference, SARC-F, and SARC-CalF for possible sarcopenia screening in Indonesia

**DOI:** 10.1038/s41598-023-36585-4

**Published:** 2023-06-17

**Authors:** Stefanus G. Kandinata, Novira Widajanti, Jusri Ichwani, Hadiq Firdausi, I. G. P. S. Aryana, Firas F. Alkaff

**Affiliations:** 1 Faculty of Medicine, Universitas Airlangga, Surabaya, Indonesia; 2 Division of Geriatrics, Department of Internal Medicine, Dr. Soetomo General Academic Hospital – Faculty of Medicine Universitas Airlangga, Jl. Mayjen Prof. Dr. Moestopo No 6-8, Surabaya, Indonesia; 3 Division of Geriatrics, Department of Internal Medicine, Faculty of Medicine, Universitas Udayana, Sanglah Teaching Hospital, Bali, Indonesia; 4 Division of Pharmacology and Therapy, Department of Anatomy, Histology, and Pharmacology, Faculty of Medicine Universitas Airlangga, Jl. Mayjen Prof. Dr. Moestopo No 47, Surabaya, Indonesia; 5 Department of Internal Medicine, University Medical Center Groningen, Groningen, The Netherlands

**Keywords:** Epidemiology, Geriatrics, Diagnosis

## Abstract

Asian working group for sarcopenia (AWGS) recently introduced “possible sarcopenia” diagnosis for early identification of sarcopenia in the primary healthcare. For initial screening, 3 modalities, i.e. calf circumference (CC) measurement, strength, assistance with walking, rising from a chair, climbing stairs, and falls (SARC-F) questionnaire, and a combination of both (SARC-CalF), are recommended. However, no validation study has been done until now. Therefore, this study aims to evaluate the diagnostic performance of the recommended screening modalities using data from Indonesia. This cross-sectional study included subjects aged ≥ 60 years old who visited primary healthcare in Surabaya, Indonesia. The diagnosis of possible sarcopenia was confirmed with hand-grip strength and repeated chair stand test. Receiver operating characteristic curve analysis was used to evaluate the diagnostic performance. Among 266 subjects, 186 (70%) were diagnosed with possible sarcopenia. Using the recommended cut-off, the area under the curve, sensitivity, and specificity were 0.511, 48.39% and 53.75% for CC, 0.543, 8.60% and 100% for SARC-F, and 0.572, 19.35% and 95% for SACRC-CalF. Our findings indicate that the diagnostic performance of the recommended screening modalities is poor. Multicenter studies from different areas in Indonesia should be done to confirm these findings.

## Introduction

Sarcopenia is a prevalent geriatric syndrome that is characterized by the gradual loss of skeletal muscle mass and strength. It is associated with higher healthcare expenditure and adverse health consequences such as impaired quality of life and increased risk of morbidity and mortality^1^. However, despite the wide impact of this syndrome, there is currently no universal diagnostic criterion for the diagnosis of sarcopenia. The most commonly used diagnostic classification was developed by the European Working Group on Sarcopenia in Older People (EWGSOP) and the Asian Working Group for Sarcopenia (AWGS)^2^. Both EWGSOP and AWGS used the same measurements, i.e. muscle mass, muscle strength, and gait speed, but with different cut-off scores. It is because there are differences in ethnicities, body size, lifestyles, and cultural background between European and Asian population^3^. Depending on the diagnostic classification used, the latest systematic review and meta-analyses reported that the global prevalence of sarcopenia varied between 10 and 27%^2^.

The impact of sarcopenia in Asia is considered to be stronger than in other continents because it is the most populated and fastest aging continents in the world^3^. Therefore, AWGS introduced “possible sarcopenia” terminology in their latest guideline. It refers to a condition of reduced muscle strength with or without reduction of physical performance^4^. This new terminology is intended for early identification of people with or at risk for sarcopenia in the primary healthcare or community preventive service settings, where advanced diagnostic equipment is not available. With early identification, immediate interventions such as lifestyle interventions can be given while the patients await for confirmatory diagnosis in the secondary or tertiary healthcare centers^4^. For initial screening of possible sarcopenia, 3 modalities, i.e. the measurement of calf circumference (CC), strength, assistance with walking, rising from a chair, climbing stairs, and falls (SARC-F) questionnaire, and a combination of both (SARC-CalF) are recommended. When the screening evaluation gives an indication for possible sarcopenia, muscle strength and physical performance assessment are done to confirm the possible sarcopenia diagnosis^4^.

While the purpose for simple initial screening in primary healthcare settings are deemed beneficial, the cut-off scores determination for the screening modalities are based on the research evidences from only 7 Asian countries, i.e. China, Hong Kong, Japan, Singapore, South Korea, Taiwan, and Thailand^4^. Within the Asia continent, there is a wide range of ethnicities, cultural, lifestyle, and socioeconomic variations^3^, and this could possibly influence the applicability of the screening modalities. However, to the best of our knowledge, no validation study using data from other Asian countries has been done to confirm the diagnostic performance of the recommended screening modalities. Therefore, there is an urgent need for a validation study.

For a validation study, the use of data from Indonesian population might be of importance. There are 3 main reasons for this. First, Indonesia has the highest number of elderly population among other countries in Southeast Asia region (account for 30.86% of all elderly population in South East Asia region), and the fourth highest in Asia continent after China, India, and Japan^5^. Second, using the same diagnostic classification, the prevalence of sarcopenia in Indonesia was 50.25%^6^, higher than the average prevalence in Asian population that was reported to be 14%^3^. Third, Indonesian population have significantly smaller body size than Singaporean population^7^; however, the cut-off scores for the screening modalities were formulated based on the data from Singaporean and not from the Indonesian population^4^. Therefore, this study aims to evaluate the diagnostic performance of 3 recommended modalities, i.e. CC, SARC-F, and SARC-CalF, for possible sarcopenia screening using data from Indonesian population.

## Methods

### Design and study population

We used data from our previously published cross-sectional study^8^. This study was conducted in Surabaya city, the capital city of East Java province, Indonesia, between December 2017 and March 2018. Surabaya city had 63 primary healthcare centers across 5 administrative regions. We randomly selected 1 primary healthcare center from each administrative regions (5 primary healthcare centers in total) for the study site. The study population were subjects aged ≥ 60 years old who visited one of the selected primary healthcare centers during the study period^8^. Total sampling method was used in this study. Inclusion criteria were subjects who are willing to participate in the study and signed the inform consent. Exclusion criteria were subjects with severe cardiovascular or respiratory diseases, history of pacemaker implantation, and impaired cognitive function. However, we used the AWGS 2014 guideline for the previous study^3^, whereas this study used the AWGS 2019 guideline^4^. The study was approved by the Ethical Committee of the Faculty of Medicine Universitas Airlangga prior to the data collection (Approval number: 273/EC/KEPK/FKUA/2017) and conducted in accordance with the Declaration of Helsinki.

The minimum required sample size was calculated using EpiInfo^9^. According to the data from the Bureau of Statistics, the total number of elderly in Surabaya city was 246,069 in 2018^10^. Using the prevalence of sarcopenia from our previous finding (41.8%)^8^, with a confidence level of 95%, and precision of 7%, a minimum of 191 subjects were needed to obtain sufficient statistical power.

### Study instruments

This study used primary data collection. Sociodemographic and clinical characteristics were collected using a questionnaire. Cognitive function was evaluated using abbreviated mental test (AMT), where score below 8 indicates moderate cognitive impairment and were subjected for exclusion^11^. Nutritional state was assessed using the Mini Nutritional Assessment (MNA).

For possible sarcopenia screening, all 3 modalities, CC, SARC-F, and SARC-CalF, were evaluated. CC is measured with the subjects in a sitting condition using a non-elastic tape to find the maximum circumference of both calves. The SARC-F questionnaire evaluates 5 components: strength, walking assistance, rising from a chair, stair-climbing, and falls. Each component is given a score of 0–2. SARC-CalF is a combination between SARC-F and CC. The scoring of SARC-CalF is similar to SARC-F; however, additional 10 points is given to male subjects having CC < 34 cm or female subjects having CC < 33 cm. The cut-off scores for the possible sarcopenia based on the AWGS 2019 guideline are as follows: CC < 34 cm for male subjects or CC < 33 cm for female subjects, SARC-F score ≥ 4, or SARC-CalF score ≥ 11^4^.

To confirm the diagnosis of possible sarcopenia, 2 indicators were used based on AWGS 2019 guideline, i.e. muscle strength and physical performance. The muscle strength was measured by TKK 5001 Grip A dynamometer, while physical performance was measured by the repeated chair stand test. The diagnosis of possible sarcopenia was confirmed if one of the indicators was fulfilled: (1) handgrip strength of < 28 kg for male subjects and < 18 kg for female subjects; (2) repeated chair stand test ≥ 12 s^4^.

### Data analyses

Data were analyzed using SPSS statistics version 25.0 for Windows (IBM Corp., Armonk, NY, USA). Continuous variables were presented as median [interquartile range], and categorical variables were presented as frequency (percentages). Sensitivity, specificity, positive predictive value (PPV), negative predictive value (NPV), and likelihood ratio (LR) were calculated and receiver operating characteristic (ROC) curve analysis was performed to evaluate the diagnostic performance of each screening modalities in predicting possible sarcopenia. In all analyses, p-value < 0.05 was considered statistically significant.

## Results

**Figure 1:**
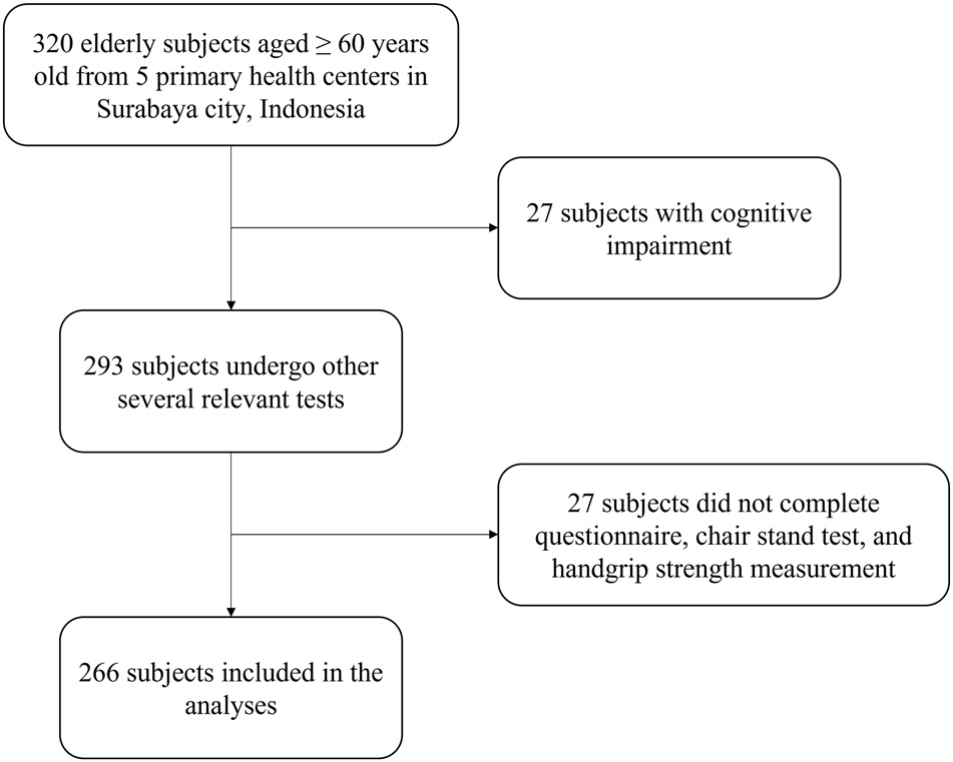
Flow chart of the study population selection.

There were 320 subjects from five primary healthcare centers in Surabaya screened for the study. Of them, 27 subjects were excluded because of cognitive impairment and 27 other subjects were excluded because they did not finished the relevant tests needed for this study, leaving 266 subjects for the analyses (Fig. 1). The majority of subjects (71.8%) were female, with median age of 65 years. More than half of the study subjects undergone routine exercise and were in good nutritional state. Detailed clinical characteristics of the study subjects are presented in Table 1.

**Table 1:** Study subjects’ clinical characteristics.

Variable	Total (N = 266)	Female (N = 191)	Male (N = 75)
Age, years	65 [61–70]	63 [60–68]	68 [63–74]
Body mass index, Kg/m^2^	22.6 [20–25.6]	23.3 [20.4–26.1]	21.7 [19.2–23.6]
Educational background, n (%)			
Up to high school graduates	252 (94.74)	180 (94.24)	72 (96)
University graduates	14 (5.26)	11 (5.76)	3 (4)
Monthly income, n (%)			
Below minimum wage	248 (93.2)	179 (93.7)	69 (92)
Above minimum wage	18 (6.8)	12 (6.3)	6 (8)
Walking assistance, n (%)			
Without a walking device	260 (97.74)	186 (97.38)	74 (98.67)
With a walking device	6 (2.26)	5 (2.62)	1 (1.33)
Routine physical exercise, n (%)			
No	93 (34.96)	64 (33.51)	29 (38.67)
Yes	173 (65.04)	127 (66.49)	46 (61.33)
Nutritional state, n (%)			
Normal	194 (72.93)	140 (73.3)	54 (72)
In risk for malnutrition	68 (25.56)	47 (24.6)	21 (28)
Malnutrition	4 (1.5)	4 (2.09)	0 (0)

**Figure 2:**
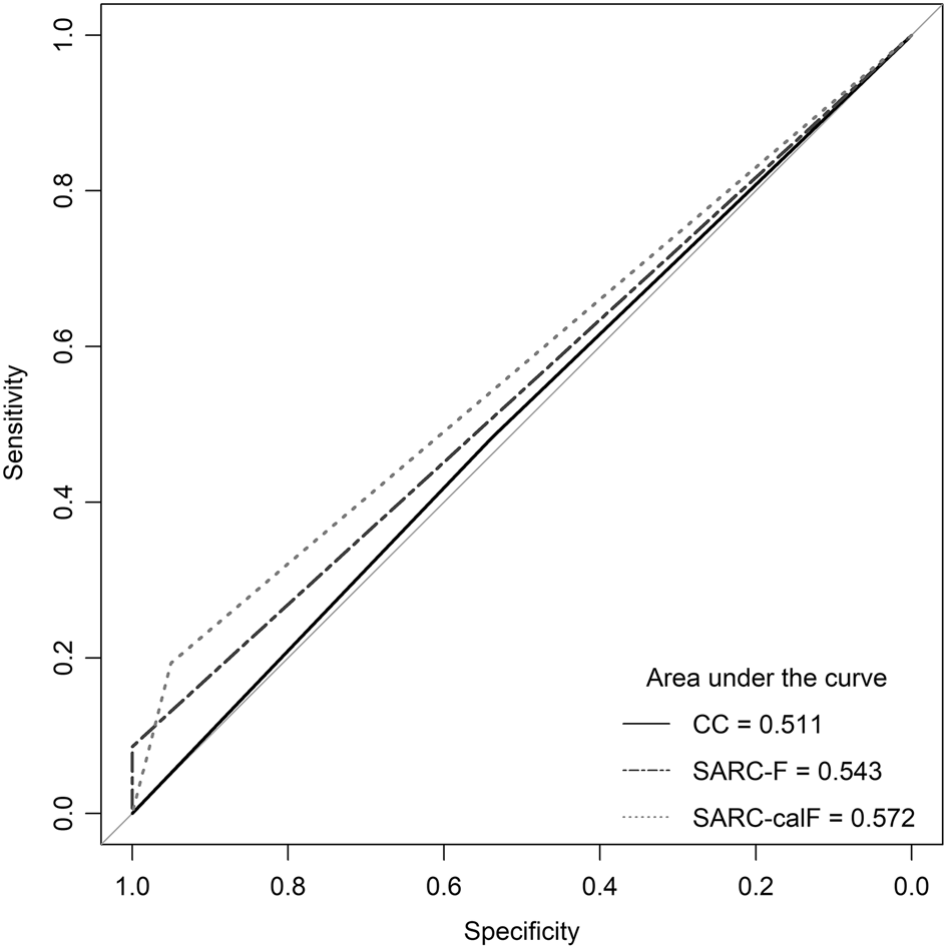
Receiver operating characteristic curve of CC, SARC-F, and SARC-CalF using the recommended cut-off from the AWGS 2019 guideline for possible sarcopenia screening. *CC* calf-circumference measurement, *SARC*-F strength, assistance with walking, rising from a chair, climbing stairs, and falls questionnaire, *SARC*-*CalF* combination of SARC-F and CC.

From 266 subjects, 186 (69.9%) of them were confirmed for possible sarcopenia from either having low handgrip strength or prolonged repeated chair stand test. When CC measurement was used as the screening modality, 127 (47.7%) subjects were suggestive of possible sarcopenia. In comparison, only 16 (6%) subjects were suggestive of possible sarcopenia when using SARC-F and 40 (15%) subjects when using SARC-CalF. CC had the highest sensitivity, whereas SARC-F had the highest specificity. The area under the curve (AUC) was 0.511 for CC, 0.543 for SARC-F, and 0.572 for SARC-CalF. The detailed diagnostic performance are presented in Table 2. The visualization of the ROC curve is shown in Fig. 2.

**Table 2:** Diagnostic performance of CC, SARC-F and SARC-CalF for possible sarcopenia screening.

Screening modalities	N	Confirmed possible sarcopenia		Sensitivity	Specificity	PPV	NPV	LR +	LR–	AUC (95% CI)	p-value
		Yes (N = 168)	No (N = 80)								
CC											
M < 34 cm, F < 33 cm	127	90	37	48.39%	53.75%	70.87%	30.94%	1.05	0.96	0.511 (0.435–0.586)	0.782
M ≥ 34 cm, F ≥ 33 cm	139	96	43								
SARC-F											
≥ 4	16	16	0	8.60%	100%	100%	68%	∞	0.91	0.543 (0.470–0.616)	0.266
< 4	250	170	80								
SARC-CalF											
≥ 11	40	36	4	19.35%	95%	90%	33.63%	3.87	0.85	0.572 (0.500–0.643)	0.063
< 11	226	150	76								

*95%CI* 95% confidence interval, *AUC* area under the operating curve, *CC* calf circumference, *NPV* negative predictive value, *PPV* positive predictive value, *LR* likelihood ratio, *SARC*-*F* Strength, assistance with walking, rising from a chair, climbing stairs, and falls, *SARC*-*CalF* combination of SARC-F and CC.

## Discussion

In this study, we evaluated the diagnostic performance of 3 modalities for possible sarcopenia screening that is recommended by the AWGS 2019 guideline, including the cut-off value. Our study showed that the diagnostic performance of the recommended modalities were poor, as indicated by the low sensitivity and low AUC.

In theory, diagnostic performance of a screening modality is expected to have high sensitivity, specificity, PPV, and PPV, and an AUC nearing 1.0. However, the chance of finding such screening modality is very small. Therefore, depending on the expected performance, screening modality in practice do not necessarily need to be highly sensitive and specific at the same time. If the intended use is to screen disease with the low prevalence, high specificity and PPV are more favorable. In contrast, greater emphasis should be given towards sensitivity and NPV if it is intended to screen disease with high prevalence^12^. Since sarcopenia is common, screening modality should have high sensitivity and NPV.

Using the cut-off from AWGS 2019^4^, we found that the CC had the highest sensitivity rate but lowest specificity rate among the screening indicators, although both are relatively low. This study’s finding is similar to the Korean study by Kim and Won^13^ which found that CC as the screening indicator had higher sensitivity (62.9–76.3%) but lower specificity (57.5–59.7%) in detecting sarcopenia compared to SARC-F and SARC-CalF^13^. Chinese study by Mo et al.^14^ found higher sensitivity and specificity of CC compared to our study and the Korean study (81.4% sensitivity and 77.0% specificity)^14^. Considering that our study, Chinese study, and Korean study used the same AWGS 2019 cut-off for CC with variable results of accuracy, it is suggested that there is a variability of the accuracy of CC as the screening indicator for possible sarcopenia, possibly influenced by factors such as anthropometry, nutritional, or others.

Both SARC-F and SARC-CalF in this study had comparable specificity; however, the sensitivity was higher in SARC-CalF. Similar finding has been shown by Ito et al.^15^, where the sensitivity of SARC-CalF was higher than SARC-F^15^. The low sensitivity of both indicators deems them unsuitable to be used for screening, but their high level of specificity makes them favorable to be used in diagnosing possible sarcopenia.

In this study, we found that CC had the lowest AUC and SARC-CalF had the highest AUC. Our findings were in contrast with previously published studies from Korea^13^ and China^14^, where both of the studies found that the CC has a relatively higher accuracy based on the AUC value compared to either SARC-F or SARC-CalF. The establishment of CC as a screening indicator of sarcopenia stems from the understanding that CC reflects the muscle mass component of sarcopenia pathophysiology^16^. However, there are functional aspects of sarcopenia that cannot be reflected by CC^17^. Considering this issue, and another finding by Ito et al.^15^ that establishes no significant change of accuracy of either CC, SARC-F, or SARC-CalF before and after change in AWGS cut-off^15^, we postulate that CC is specifically less accurate in detecting condition defined by AWGS 2019 guideline as “possible sarcopenia”, in which physical characteristic of sarcopenia does not always profound in the patients.

Other than that, some factors possibly influence the CC measurement. For instance, edema in the calf possibly overstates the muscular volume of the calf, influencing the accuracy of CC as a screening indicator of sarcopenia^18^. There is also a possible influence of the difference in CC measurement method in some studies, specifically in what posture the CC measurement is conducted. Ito et al.^15^, for example, conducted the CC measurement while the subject was in a sitting position, similar to our study^15^. Meanwhile, both Kusaka et al.^19^ and Kawakami et al.^20^ measured the CC while the subject was in standing and supine, respectively^19,20^. Those 3 studies provided different accuracies of CC, with sensitivity ranging from 73.6 to 88.0% and specificity ranging from 62.8 to 91.0%. With some studies finding the significant influence of posture on CC measurement^21,22^, standardization is needed to ensure the reliability of CC as the screening indicator for possible sarcopenia. For instance, since some studies found that CC measurement in a standing position has higher accuracy and lower overestimation^21,23^, we need to consider standardizing the CC measurement in a standing position.

There are several limitations in this study. This study only includes subjects from one urban city; thus, generalization of this study’s findings should be done cautiously. The majority of the subjects were women, whereas it is known that gender plays an important role in sarcopenia^24,25^. Next to that, we did not record any subjects with pretibial edema conditions. Nonetheless, this is the first study that validate the diagnostic performance of 3 screening indicators proposed by the AWGS 2019 guideline to detect possible sarcopenia, a definition that was also introduced by the AWGS in the same year^4^.

## Conclusion

Our findings showed that none of the screening modalities that were recommended by the AWGS in their latest guideline were able to identify elderly subjects for possible sarcopenia in our study population. This suggest that the current cut-off scores for initial screening for possible sarcopenia may not be applicable in Indonesia. Multicenter studies with larger study population from other urban areas and also rural areas in Indonesia should be done to confirm these findings.

## Data Availability

The datasets generated during and/or analysed during the current study are available from the corresponding author on reasonable request.

## References

[1] Cruz-Jentoft AJ, Sayer AA (2019). Sarcopenia. Lancet.

[2] Petermann-Rocha F (2022). Global prevalence of sarcopenia and severe sarcopenia: A systematic review and meta-analysis. J. Cachexia Sarcopenia Muscle.

[3] Chen LK (2014). Sarcopenia in Asia: Consensus report of the Asian Working Group for Sarcopenia. J. Am. Med. Dir. Assoc..

[4] Chen LK (2020). Asian Working Group for Sarcopenia: 2019 consensus update on sarcopenia diagnosis and treatment. J. Am. Med. Dir. Assoc..

[5] United Nations. Department of Economic and Social Affairs. Population Division. World Population Ageing 2019: Highlight (2019).

[6] Ridwan ES (2021). Peak expiratory flow rate and sarcopenia risk in older Indonesian people: A nationwide survey. PLoS One.

[7] Chuan TK, Hartono M, Kumar N (2010). Anthropometry of the Singaporean and Indonesian populations. Int. J. Ind. Ergon..

[8] Widajanti N (2020). Sarcopenia and frailty profile in the elderly community of surabaya: A descriptive study. Acta Med. Indones..

[9] Epi Info™ (2011). A Database and Statistics Program for Public Health Professionals.

[10] Badan Pusat Statistik Kota Surabaya. *Proyeksi Penduduk Kota Surabaya (Jiwa)*. https://surabayakota.bps.go.id/indicator/12/197/1/proyeksi-penduduk-kota-surabaya.html (2018).

[11] Jitapunkul S, Pillay I, Ebrahim S (1991). The abbreviated mental test: Its use and validity. Age Ageing.

[12] FDA-NIH Biomarker Working Group. In *BEST (Biomarkers, EndpointS, and other Tools) Resource* (2016). 27010052

[13] Kim M, Won CW (2020). Sarcopenia in Korean community-dwelling adults aged 70 years and older: Application of screening and diagnostic tools from the Asian working group for Sarcopenia 2019 update. J. Am. Med. Dir. Assoc..

[14] Mo YH (2021). Comparison of three screening methods for sarcopenia in community-dwelling older persons. J. Am. Med. Dir. Assoc..

[15] Ito A (2021). Changes in the screening efficacy of lower calf circumference, SARC-F score, and SARC-CalF score following update from AWGS 2014 to 2019 sarcopenia diagnostic criteria in community-dwelling older adults. J. Phys. Ther. Sci..

[16] Chen CY (2020). Calf circumference as an optimal choice of four screening tools for sarcopenia among ethnic chinese older adults in assisted living. Clin. Interv. Aging.

[17] Rolland Y (2003). Sarcopenia, calf circumference, and physical function of elderly women: A cross-sectional study. J. Am. Geriatr. Soc..

[18] Ishida Y (2019). Impact of edema on length of calf circumference in older adults. Geriatr. Gerontol. Int..

[19] Kusaka S (2017). Thigh and calf circumference for the sarcopenia screening in community-dwelling elderly women. J. Clin. Gerontol. Geriatr..

[20] Kawakami R (2015). Calf circumference as a surrogate marker of muscle mass for diagnosing sarcopenia in Japanese men and women. Geriatr. Gerontol. Int..

[21] Jeong E, Kim M, Won CW (2020). Effects of posture, side and dominant hand on calf circumference measurement in community-dwelling older adults. Geriatr. Gerontol. Int..

[22] Sousa AS, de Sousa OL, Amaral TF (2014). The effect of posture on body circumferences in older adults. J. Hum. Nutr. Diet..

[23] Piodena-Aportadera MRB (2022). Calf circumference measurement protocols for sarcopenia screening: Differences in agreement, convergent validity and diagnostic performance. Ann. Geriatr. Med. Res..

[24] Du Y (2019). Sex differences in the prevalence and adverse outcomes of sarcopenia and sarcopenic obesity in community dwelling elderly in East China using the AWGS criteria. BMC Endocr. Disord..

[25] Hwang J, Park S (2022). Gender-specific risk factors and prevalence for sarcopenia among community-dwelling young-old adults. Int. J. Environ. Res. Public Health.

